# Faults locating of power distribution systems based on successive PSO-GA algorithm

**DOI:** 10.1038/s41598-024-61306-w

**Published:** 2024-05-17

**Authors:** Wenzhang Xu, Jiachun Li, Lv Yang, Quan Yu

**Affiliations:** 1 School of Mechanical Engineering, Guizhou University, Guiyang, 550000 Guizhou China; 2 Zunyi Long March Power Distribution Equipment Co. LTD, Zunyi, 563000 Guizhou China

**Keywords:** Power distribution network, Fault location, Particle swarm algorithm, Genetic algorithm, Hybrid algorithm, Information technology, Electrical and electronic engineering

## Abstract

As the terminal of the power system, the distribution network is the main area where failures occur. In addition, with the integration of distributed generation, the traditional distribution network becomes more complex, rendering the conventional fault location algorithms based on a single power supply obsolete. Therefore, it is necessary to seek a new algorithm to locate the fault of the distributed power distribution network. In existing fault localization algorithms for distribution networks, since there are only two states of line faults, which can usually be represented by 0 and 1, most algorithms use discrete algorithms with this characteristic for iterative optimization. Therefore, this paper combines the advantages of the particle swarm algorithm and genetic algorithm and uses continuous real numbers for iteration to construct a successive particle swarm genetic algorithm (SPSO-GA) different from previous algorithms. The accuracy, speed, and fault tolerance of SPSO-GA, discrete particle swarm Genetic algorithm, and artificial fish swarm algorithm are compared in an IEEE33-node distribution network with the distributed power supply. The simulation results show that the SPSO-GA algorithm has high optimization accuracy and stability for single, double, or triple faults. Furthermore, SPSO-GA has a rapid convergence velocity, requires fewer particles, and can locate the fault segment accurately for the distribution network containing distorted information.

## Introduction

As the terminal of the power system, the distribution network is characterized by many branches, variable operation modes, a large number of devices, and poor operation conditions, which makes it the main area where distribution system failures occur. With the access of distributed generation, the traditional distribution network has changed from a single-power radiant structure to a multi-power structure, and the traditional fault location algorithm based on single-power supply is no longer applicable, so further exploring new fault location methods is necessary. In addition, due to the installation characteristics of the feeder terminal unit (FTU), the information uploaded by FTU may be distorted. Therefore, the fault location algorithm should also meet the requirements of positioning accuracy and high fault tolerance^1^.

At present, the fault location methods used in distributed power distribution networks include intelligent algorithms^2,3^, matrix location method^4^, and synchronous vector method^5^. The matrix location algorithm usually needs to establish a structure matrix related to the current distribution network and then carry out specific operations with the fault information matrix to obtain the fault information of the distribution network. Literature^6^ proposes a fault location model combining matrix and particle swarm optimization algorithms. Although the positioning accuracy and anti-interference ability are improved compared with the traditional matrix algorithm, it can also cause a dimensional disaster. The synchronous vector method can realize high-precision fault location by providing real-time current and voltage, but the synchronous vector unit (PMU) equipment is expensive^7^. Therefore, some authors^8^ have effectively mitigated this defect by considering the impact of load loss on PMU equipment layout.

In recent years, the rapid development of artificial intelligence technology has provided a reference for the solution of fault localization problems in distribution networks with multiple power structures. Among them, well-studied intelligent algorithms include particle swarm optimization (PSO)^9^, genetic algorithm (GA)^10^, artificial fish swarm algorithm (AF)^11^, and so on. However, a single PSO or GA algorithm often tends to fall into the dilemma of local optimal solution^12^. For this reason, some researchers^13^ proposed a switching function expression for multiple faults of multiple power sources and utilized a hybrid discrete PSO-GA algorithm better to solve the fault localization problem in distribution networks. However, the algorithm needs to improve its iteration speed triggered by the unsuccessful speed update, and it is prone to falling into the local optimum. Although the binary artificial fish swarm algorithm has an excellent performance in localization effect, the algorithm is complex, and the optimality-seeking speed is slow. In contrast, the continuous particle swarm algorithm can overcome the problem of speed update failure in binary iterative optimization search and has a high iteration speed. Therefore, successive particle swarm genetic algorithm (SPSO-GA) is established in this paper and takes the IEEE 33-node distributed distribution network as an example to compare and study the performances of SPSO-GA, PSO-GA, and AF in terms of localization accuracy, arithmetic speed, and fault tolerance.

## Distribution network fault location rules

### State encoding style

There are only fault and normal states in the feeder segment of the distribution network. Therefore, a 1 or 0 code can represent a specific segment's feeder state. The expression is as follows:

1 $${\text{X}}_{{\text{i}}} = \left\{ {\begin{array}{*{20}l} {1,} \hfill & {{\text{segment}}\;{\text{i}}\;{\text{is}}\;{\text{faluty}}} \hfill \\ {0,} \hfill & {{\text{segment}}\;{\text{i}}\;{\text{is}}\;{\text{not}}\;{\text{faluty}}} \hfill \\ \end{array} } \right.$$

After the DG is connected, the feeder current direction changes to bidirectional. When a fault occurs, the FTU at the node can detect the fault current, and the node status code can be expressed as:

2 $${\text{I}}_{{\text{j}}} = \left\{ {\begin{array}{*{20}l} {1,} \hfill & {{\text{Node}}\;{\text{j }}\;{\text{detects}}\;{\text{fault}}\;{\text{current}}\;{\text{in}}\;{\text{the}}\;{\text{positive}}\;{\text{direction}}} \hfill \\ { - 1,} \hfill & {{\text{Node}}\;{\text{j}}\;{\text{detects}}\;{\text{fault}}\;{\text{current}}\;{\text{in}}\;{\text{the}}\;{\text{negative}}\;{\text{direction}}} \hfill \\ {0,} \hfill & {{\text{No}}\;{\text{fault}}\;{\text{current}}\;{\text{is}}\;{\text{detected}}\;{\text{at}}\;{\text{node}}\;{\text{j}}} \hfill \\ \end{array} } \right.$$

### Switching function

The switch function expresses the connection between switch information and each feeder segment. The switch function in the distribution network containing DG is:

3 $${\text{I}}_{{\text{j}}}^{*} \left( {\text{x}} \right) = {\text{I}}_{{{\text{ju}}}} \left( {\text{x}} \right) - {\text{I}}_{{{\text{jd}}}} \left( {\text{x}} \right)$$

where

4 $${\text{I}}_{{{\text{ju}}}} \left( {\text{x}} \right) = \left\{ {\mathop \prod \limits_{{\text{u}}}^{{{\text{M}}_{1} }} \left[ {{\text{K}}_{{\text{u}}} \left( {1 - \prod {\text{X}}_{{{\text{j}},{\text{s}}_{{\text{u}}} }} } \right)} \right]} \right\} \times \mathop \prod \limits_{{{\text{j}},{\text{d}}}}^{{\text{N}}} {\text{X}}_{{{\text{j}},{\text{d}}}}$$

5 $${\text{I}}_{{{\text{jd}}}} \left( {\text{x}} \right) = \left\{ {\mathop \prod \limits_{{\text{d}}}^{{{\text{N}}_{1} }} \left[ {{\text{K}}_{{\text{d}}} \left( {1 - \prod {\text{X}}_{{{\text{j}},{\text{s}}_{{\text{d}}} }} } \right)} \right]} \right\} \times \mathop \prod \limits_{{{\text{j}},{\text{u}}}}^{{\text{M}}} {\text{X}}_{{{\text{j}},{\text{u}}}}$$

where switch j is the breaking point, the distribution network is divided into uptream and downstream, i.e., u and d two parts, the upstream and downstream lines have M1 and N1 power supplies respectively; “$\prod$” means “logical or”; ${I}_{j}^{*}\left(x\right)$ is the switch function of the jth switch, ${I}_{ju}\left(x\right)$ and ${I}_{jd}\left(x\right)$ are the switching functions of the upstream and downstream lines of the jth switch; ${K}_{u}$ and ${K}_{d}$ are the power switching coefficients of the upstream and downstream lines, which are taken as 1 if the power supply is connected and 0 otherwise; ${X}_{j,Su}$ and ${X}_{j,Sd}$ are the status values of the feeder section from the jth switch to the upstream power supply ${S}_{u}$ and the downstream power supply ${S}_{d}$; ${X}_{j,u}$ and ${X}_{j,d}$ are the states of the upstream and downstream feeder sections, respectively; M and N are the total number of feeder sections of the upstream and downstream lines, respectively.

### Fitness function

The fitness function is a function used to compare ideal state value and actual value, and its expression is as follows^14^:

6 $${\text{f}}\left( {{\text{x}}_{{\text{j}}} } \right) = \mathop \sum \limits_{{{\text{j}} = 1}}^{{\text{n}}} \left| {{\text{I}}_{{\text{j}}} - {\text{I}}_{{\text{j}}}^{*} } \right| + {\upomega }\mathop \sum \limits_{{{\text{j}} = 1}}^{{\text{n}}} {\text{X}}_{{\text{j}}}$$

where $I_{j}$ is the actual reported information of FTU at switch j, and ${{I}^{*}}_{j}$ is the expected status value of FTU at switch j; n is the total number of extents and nodes; ${X}_{j}$ represents the state of the j section; ω is the anti-misjudgment weight coefficient. The second term on the right can solve the case of multiple solutions of the function. The smaller the fitness function value, the closer the predicted fault segment is to the actual fault segment.

## Distribution network fault location algorithm

### Improvement of particle swarm optimization algorithm

The particle swarm optimization model is built to imitate the foraging process of birds^15^, and its binary particle speed and position update expression is as follows:

7 $${\text{v}}_{{{\text{id}}}} \left( {{\text{t}} + 1} \right) = {\upomega v}_{{{\text{id}}}} \left( {\text{t}} \right) + {\text{c}}_{1} {\text{r}}_{1} \left( {{\text{pbest}}_{{{\text{id}}}} \left( {\text{t}} \right) - {\text{x}}_{{{\text{id}}}} \left( {\text{t}} \right)} \right) + {\text{c}}_{2} {\text{r}}_{2} \left( {{\text{gbest}}_{{{\text{id}}}} \left( {\text{t}} \right) - {\text{x}}_{{{\text{id}}}} \left( {\text{t}} \right)} \right)$$

8 $${\text{x}}_{{{\text{id}}}} \left( {{\text{t}} + 1} \right) = \left\{ {\begin{array}{*{20}l} {1,} \hfill & {{\text{rand}} < {\text{sign}}\left( {{\text{v}}_{{{\text{id}}}} \left( {{\text{t}} + 1} \right)} \right)} \hfill \\ {0,} \hfill & {{\text{other}}} \hfill \\ \end{array} } \right.$$

9 $${\text{sign}}\left( {{\text{v}}_{{{\text{id}}}} \left( {{\text{t}} + 1} \right)} \right) = \left\{ {\begin{array}{*{20}l} {0.98,} \hfill & {4 < {\text{v}}_{{{\text{id}}}} \left( {{\text{t}} + 1} \right)} \hfill \\ {\frac{1}{{1 + {\text{e}}^{{ - {\text{v}}_{{{\text{id}}}} \left( {{\text{t}} + 1} \right){ }}} }},} \hfill & { - \,4 \le {\text{v}}_{{{\text{id}}}} \left( {{\text{t}} + 1} \right) \le 4} \hfill \\ { - \,0.98, } \hfill & {{\text{v}}_{{{\text{id}}}} \left( {{\text{t}} + 1} \right) < - \,4} \hfill \\ \end{array} } \right.$$

In the formula, ${v}_{id}$ and ${x}_{id}$ are the velocity and position of the d dimension of the ith particle, respectively. ω is the inertia weight; ${c}_{1}$ and ${c}_{2}$ are learning factors; ${r}_{1}$ and ${r}_{2}$ are random numbers; $t$ is the number of iterations; ${gbest}_{id}\left(t\right)$ is the d-dimensional position of the global optimal particle in the t-th iteration, and ${pbest}_{id}\left(t\right)$ is the d-dimensional position of the historical optimal particle i in the t-th iteration.

By observing the second and third items on the right of Eq. (7), it can be seen that there is a 50% probability that this item will be zero, which will lead to the failure of speed updating and will not improve with the increase of iterations, significantly weakening the convergence speed and optimization ability of the algorithm. Therefore, the coding method of “dual structure coding of particle position” proposed in the literature^16^ is introduced in this paper to construct successive particle swarm optimization (SPSO) for fault location of the distribution network, which is defined as follows:

10 $${\text{v}}_{{{\text{id}}}} \left( {{\text{t}} + 1} \right) = {\upomega v}_{{{\text{id}}}} \left( {\text{t}} \right) + {\text{c}}_{1} {\text{r}}_{1} \left( {\overline{{{\text{pbest}}_{{{\text{id}}}} }} \left( {\text{t}} \right) - \overline{{{\text{x}}_{{{\text{id}}}} }} \left( {\text{t}} \right)} \right) + {\text{c}}_{2} {\text{r}}_{2} \left( {\overline{{{\text{gbest}}_{{{\text{id}}}} }} \left( {\text{t}} \right) - \overline{{{\text{x}}_{{{\text{id}}}} }} \left( {\text{t}} \right)} \right)$$

11 $$\overline{{{\text{x}}_{{{\text{id}}}} }} \left( {{\text{t}} + 1} \right) = \overline{{{\text{x}}_{{{\text{id}}}} }} \left( {\text{t}} \right) + {\text{v}}_{{{\text{id}}}} \left( {{\text{t}} + 1} \right)$$

12 $${\text{x}}_{{{\text{id}}}} \left( {{\text{t}} + 1} \right) = \left\{ {\begin{array}{*{20}l} {1,} \hfill & {0.5 < {\text{sign}}\left( {\overline{{{\text{x}}_{{{\text{id}}}} }} \left( {{\text{t}} + 1} \right)} \right)} \hfill \\ {0,} \hfill & {{\text{other}}} \hfill \\ \end{array} } \right.$$

where $sign(x)=\frac{1}{1+{e}^{-x}}$; ${v}_{id}\left(t\right)\in [-\mathrm{4.5,4.5}]$; $\overline{{gbest }_{id}}\left(t\right)$, $\overline{{pbest }_{id}}\left(t\right)$, $\overline{{x }_{id}}\left(t\right)\in \left[-5{\text{k}},5{\text{k}}\right]$. this article takes $k = 1.5$. The improved algorithm retains the advantages of the PSO algorithm in continuous space search and applies to discrete space optimization problems.

### Binary fish swarm algorithm

In a piece of water, fish can often find the place with more nutrients by themselves or with other fish, so the place with the most significant number of fish surviving is generally the place with the most nutrients in the water. The artificial fish swarm algorithm is based on this feature to achieve optimization. Artificial fish shoals are usually abstracted into four behaviors: swarm behavior, tailgating behavior, and foraging behavior. In recent years, the AF algorithm has become one that can solve binary optimization problems. In order to reflect the advantages and effectiveness of the algorithm established in this paper, the AF algorithm is introduced as a comparison, and its relevant definitions are as follows:

#### The gathering behavior

Fish naturally gather in groups to ensure their survival and avoid hazards during swimming. There are three rules for fish to abide by when they gather in groups: separation rules: try to avoid overcrowding with neighboring partners; Alignment rules: try to be consistent with the average direction of neighboring partners; Cohesion rule: Try to move toward the center of the neighboring partner.

Suppose the current state of $i$ artificial fish is ${x}_{i}$, the food concentration is ${f}_{i}$, and the number of artificial fish in its field of vision ($norm\left({x}_{i}-{x}_{j}\right)<visual$) centering on itself is ${n}_{s}$. If ${n}_{s}\ge 1$, the binary central expression is:

13 $$x_{j} = \left\{ {\begin{array}{*{20}l} {1,} \hfill & {\rho > 0.5} \hfill \\ {round\left( {rand} \right),} \hfill & {\rho = 0.5} \hfill \\ {0,} \hfill & {\rho < 0.5} \hfill \\ \end{array} } \right.$$

where $\rho = \frac{{sum\left( {x_{j} } \right)}}{{n_{f} }}$, round is the integer function.

Calculate the food concentration $f_{j}$ of the center, if satisfied:

14 $$f_{j} < f_{i} ,\;f_{j} /n_{f} < \sigma \cdot f_{i}$$

Note the center is not too crowded. Follow the formula (15) to move toward the center. Otherwise, foraging behavior is performed.

15 $$x_{{\left( {i + 1} \right)k}} = \left\{ {\begin{array}{*{20}l} {x_{jk} ,} \hfill & {select \,nn \le Step \,positions \,on{ \,}x_{j} \,at \,random} \hfill \\ {x_{ik} ,} \hfill & {other} \hfill \\ \end{array} } \right.$$

In this paper, n is obtained by the function randperm (). For Eq. (15), randperm (Step, 1) is taken here. The $Step$ is the step size of each move.

#### The rear-ending behavior

When one or more of the fish in the shoal finds food, its neighbor follows it quickly to the spot.

Suppose the current state of artificial fish $i$ is $x_{i}$, the food concentration is $f_{i}$, and $x_{j}$ is the partner with the lowest food concentration among all partners in its field of vision centered on itself. If $f_{j} > f_{i}$, foraging behavior is performed; Otherwise, use Formula (14) centered on $x_{j}$ to judge whether it is crowded. If not, move with function (15); otherwise, foraging behavior will be performed.

#### The foraging behavior

Fish generally swim freely in the water at random, and when they find food, they swim quickly toward increasing food.

Set the current state of the $i$th artificial fish as $x_{i}$ and food concentration as $f_{i}$, and select a state $x_{j}$ randomly in the field of vision according to Formula (16) with food concentration as $f_{j}$.

16 $$x_{jk} = \left\{ {\begin{array}{*{20}l} {\sim x_{ik} ,} \hfill & {select\; nn\,<\, Visual\; positions\; on\;x_{i} \;at \;random} \hfill \\ {x_{ik} , } \hfill & {other} \hfill \\ \end{array} } \right.$$

If $f_{j} < f_{i}$, move one step in this direction according to (17). The Try-number is repeated if the maximum search times cannot be met. If the maximum search times are not met, random behavior (18) is executed

17 $$x_{{\left( {i + 1} \right)k}} = \left\{ {\begin{array}{*{20}l} {x_{jk} ,} \hfill & {randomly\; select\; m\left( {m \le Step} \right)\; elements\; from \;the \;selected\; n \;positions} \hfill \\ {x_{ik} ,} \hfill & {other} \hfill \\ \end{array} } \right.$$

#### The random behavior

Individual fish swim randomly in the water to find food spots or partners around them in a broader area.

18 $$x_{{\left( {i + 1} \right)k}} = \left\{ {\begin{array}{*{20}l} {\ x_{{ik}} ,} \hfill & {select~\;l\left( {l\, \le \,Step} \right)~\;positions~\;on\;~{\text{}}x_{i} ~\,at\;~random} \hfill \\ {x_{{ik}} ,~} \hfill & {other} \hfill \\ \end{array} } \right.$$

### PSO-GA and SPSO-GA algorithms for distribution network fault location

**Figure 1:**
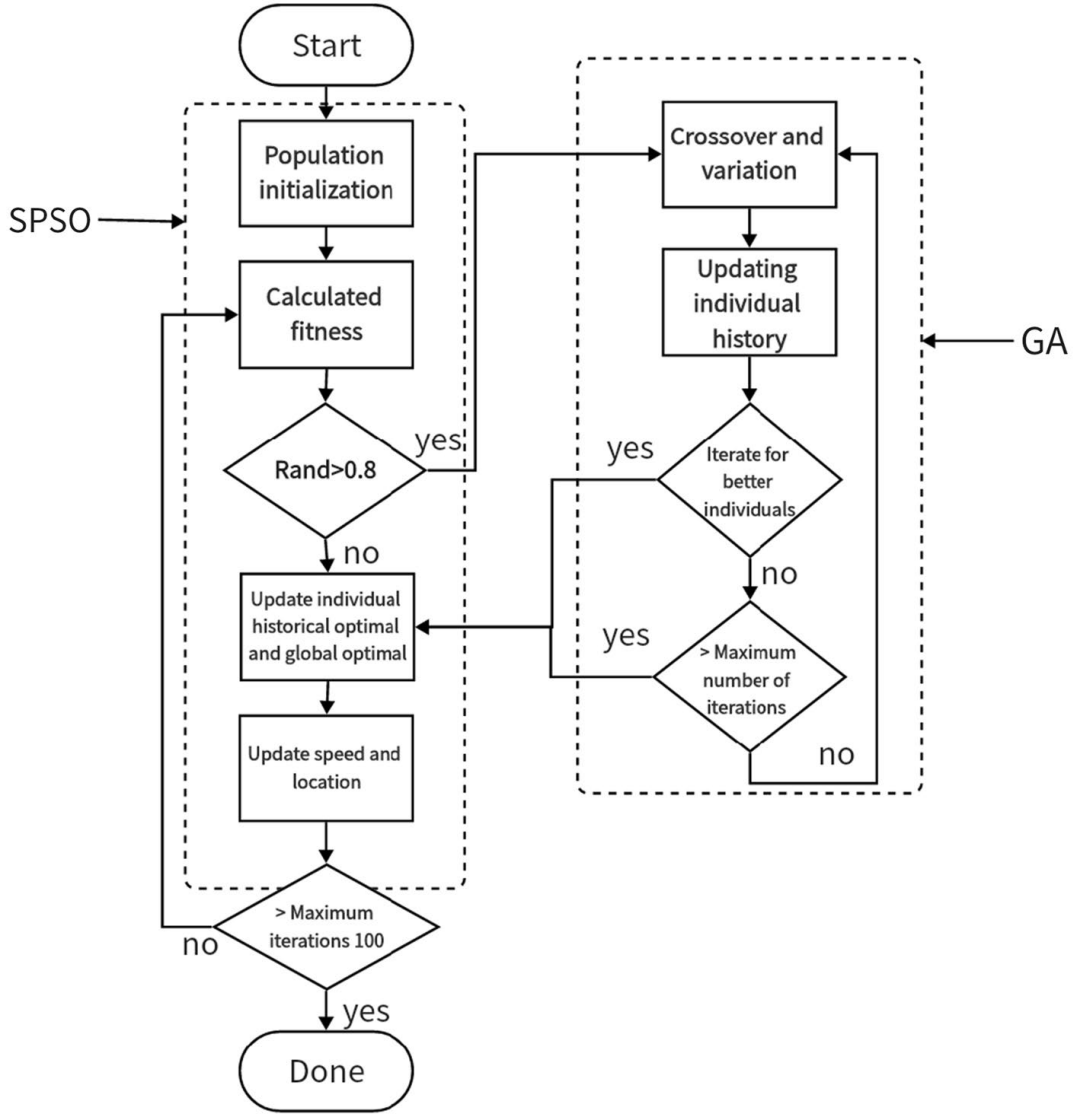
PSO-GA algorithm flowchart.

In this paper, the concepts of gene crossover and mutation in GA were introduced in the iteration of the PSO algorithm to increase the diversity of particles^17^. In this paper, the mutation effect is achieved by inverting a specific number, different from the variation in the previous binary genetic algorithm. In order to balance the computational efficiency, each iteration has a 20% probability of going through an additional the GA substep. After entering GA substep, iteration $G=30$ times, where crossover probability $pc=0.9$, mutation probability $pm=0.5$. When the GA substep gets a better individual than the global optimal of the previous generation, exit the GA substep immediately to improve the operation efficiency. The process is shown in Fig. 1.

## Comparison of fault optimization in SPSO-GA, PSO-GA and AF distribution networks

**Figure 2:**
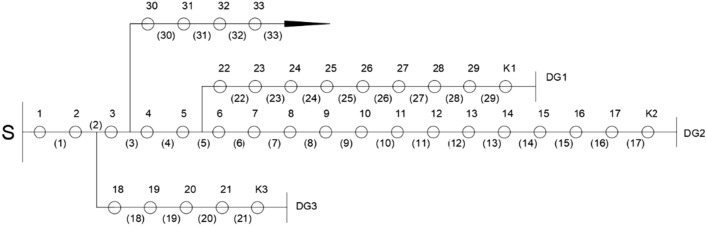
IEEE 33-node distribution network.

In order to compare and study the optimization ability of the three algorithms, this paper takes the IEEE33-node distribution network as the object. It uses MATLAB simulation to verify the algorithm's effectiveness in terms of optimization accuracy, fault tolerance, and optimization speed. The network structure of the distribution network is shown in Fig. 2:

The number of individuals of the three algorithms is set as $N=20$, the number of single iterations of the program is set as 100, and each algorithm is tested 200 times to reduce occasionality.

### Optimal parameter verification

In order to make the comparison results more convincing, it is necessary to find out the optimal parameters of each algorithm. In this paper, Section “Conclusion”, the fault of the distribution network is selected as the optimization object of the three algorithms, and the grid method is used to find the optimal parameters.

The learning factors c1 and c2 of the particle swarm optimization algorithm are generally set in the interval of [0,4], and the algorithm is more stable when the inertia weight $\omega$ is less than 1. In this paper, the parameters of the PSO-GA algorithm are set as $\omega =0.9,c1=3.4,c2=3.5$.

For the SPSO-GA algorithm proposed in this paper, The debugging steps are as follows:

1. This algorithm uses a different continuous velocity (only containing 0 and 1) for iteration, so the velocity of some particles will gradually approach 0, resulting in a slower position update. Therefore, the random velocity between (0, ± 2) is re-assigned to the particles with lower velocity to retain the positive and negative of the original velocity to prevent the particles from falling into the local optimal state.
2. Through debugging, the basic parameters of the SPSO-GA algorithm are set as $=0.8$, $c1=0.7$, $c2=0.1$.

For the AF algorithm, Step and Visual are restricted to integers in the interval (0,4], and the size of the delta is also restricted to the interval (0,4]. The parameter combination with the highest accuracy was selected, and finally, the parameters of the algorithm were set as Step = 2, visual = 4 and, delta = 2.5.

### Comparison of search accuracy

Table 1 compares the positioning accuracy obtained for each optimization-seeking algorithm across 200 experiments conducted under various human-specified fault scenarios. It can be seen from the table that the optimization accuracy of the PSO-GA algorithm and AF algorithm in different fault segments changes significantly, and the algorithm is unstable. At the same time, the algorithm proposed in this paper is stable and has a good optimization effect.

**Table 1:** Comparison of single fault location accuracy.

Algorithms	2 (%)	3 (%)	5 (%)	16 (%)	19 (%)	27 (%)	32 (%)
PSO-GA	100	92	95	98	11	70	16
AF	100	100	100	100	0	100	1
SPSO-GA	100	99	98.5	100	85	87	77.5

**Figure 3:**
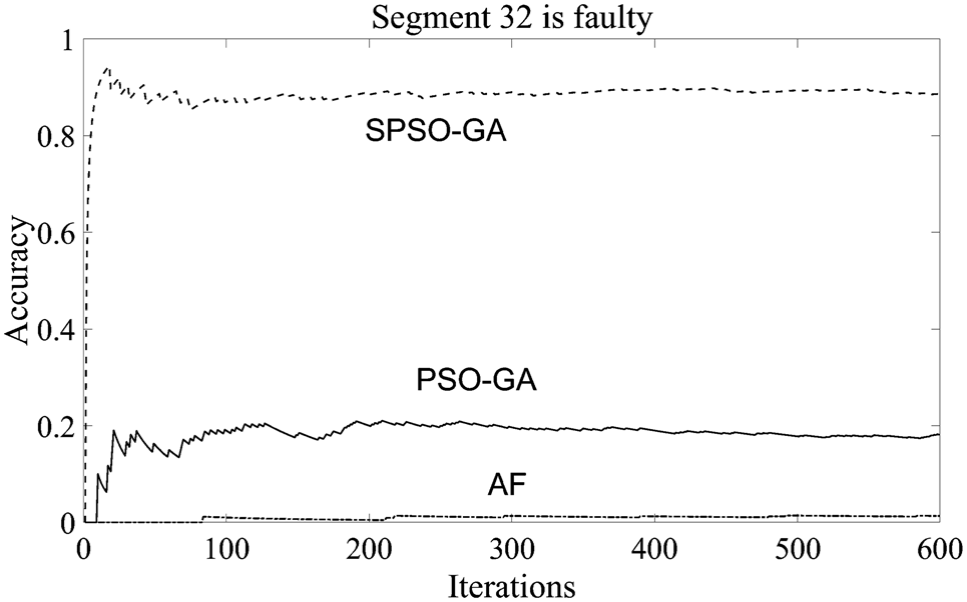
Iterative comparison after increasing the number of particles.

It can also be seen from Table 1 that when the fault occurs in section 32 of the distribution network, the optimization accuracy of the three algorithms is low; this is because there are only 20 optimization individuals set in this paper, and the probability of hybrid algorithm entering GA subroutine is only 0.2, so if we want to increase the optimization accuracy of the algorithm, in addition to increasing the number of particles in the algorithm, it can also increase the number of iterations of the GA subroutine and or increase the probability of entering the GA subroutine. Through verification, when the number of individual particles of each algorithm is increased to 30, the accuracy of the SPSO-GA algorithm constructed in this paper can reach 88%. In contrast, the optimization effect of the other two algorithms is not improved much. Figure 3 shows the iteration situation where only the particle number of each algorithm is increased to 30:

Continue to compare other fault situations. The following simulation results are still based on the particle number of the algorithm being 20, and the comparison results of dual fault location accuracy are shown in Table 2.

**Table 2:** Comparison of double fault location accuracy.

Algorithms	33|4 (%)	2|28 (%)	8|18 (%)	2|9 (%)	9|25 (%)	23|30 (%)	20|7 (%)
PSO-GA	91	100	92.5	100	96.5	26.5	85.5
AF	100	100	100	100	100	19.5	100
SPSO-GA	99	99.5	97.5	99.5	98	79.5	97.5

It can be seen from the optimization results of double faults that, except for the case of feeder segment 23 and 30 faults, all algorithms can maintain a high level in terms of stability and positioning accuracy, and the SPSO-GA algorithm has little difference with the AF algorithm in the optimization of double faults. However, the SPSO-GA algorithm is more stable in general.

Table 3 shows the optimization results of triple faults. It can be seen that when the faults co-occur in sections 15, 28, and 3, the optimization result of the AF algorithm drops significantly to only 68.5%. Although PSO-GA has a higher optimization rate in this case, the SPSO-GA algorithm shows a more stable optimization result.

**Table 3:** Comparison of accuracy of triple fault location.

Algorithms	30|6|19 (%)	20|4|27 (%)	7|5|30 (%)	1|18|4 (%)	31|26|9 (%)	22|17|19 (%)	15|28|3 (%)
PSO-GA	86.5	97.5	93.5	100	97	100	88
AF	100	100	98	100	100	100	68.5
SPSO-GA	91.5	94	92.5	100	96	100	90.5

### Comparison of fault tolerance of algorithm optimization

As the distribution network at the end of the power system, most of its faults are single faults^18^, so in order to highlight the fault tolerance of the SPSO-GA algorithm, this paper continues to simulate and compare single faults containing distorted information, and the calculation results are shown in Table 4.

**Table 4:** Comparison of single fault location accuracy with distortion information.

Algorithms	32(13) (%)	32(13|19) (%)	16(5) (%)	16(7|15) (%)
PSO-GA	9.5	11.5	96	97.5
AF	2	1.5	100	100
SPSO-GA	75.5	75	100	98.5

As seen from Table 4, the optimization accuracy of the three algorithms has a high accuracy when the fault occurs in section 16, and there is one or two distorted information. Among them, the fish swarm algorithm AF can even reach 100%. However, when 32 zone faults and distorted information exist, the optimization effect of PSO-GA and AF algorithms is significantly reduced. In contrast, the SPSO-GA algorithm shows better stability despite some changes. Therefore, the SPSO-GA algorithm can better locate the fault section with distorted information and be more stable. Similarly, if the fault optimization accuracy of the algorithm needs to be improved. In that case, it can still be achieved by adding a few particles or increasing the probability of GA substeps.

### Search for speed comparison

**Figure 4:**
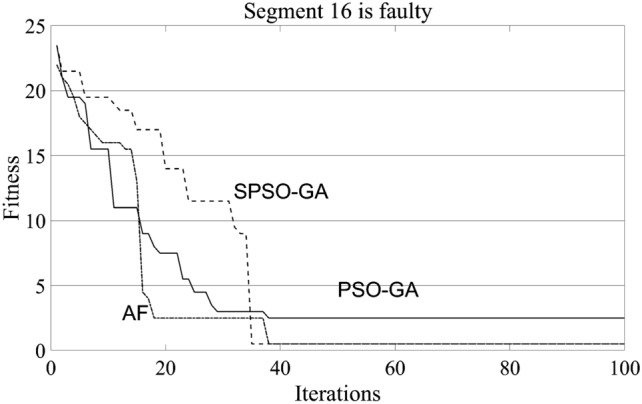
Comparison of times of single iteration.

**Figure 5:**
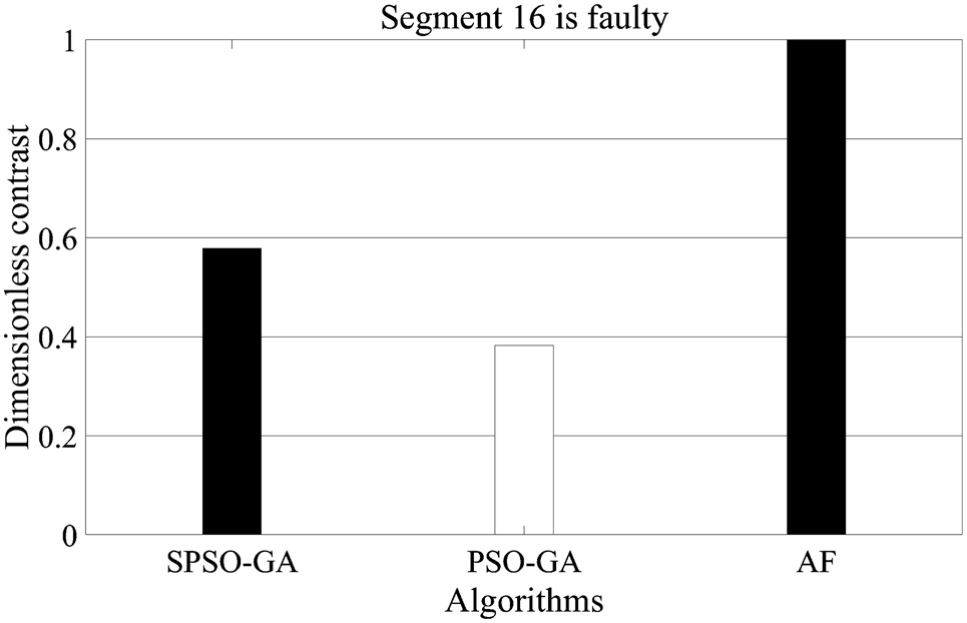
Iteration speed comparison.

Figure 4 shows the comparison results of a single iteration of the three algorithms. Since the single iteration of the algorithm is contingent, it cannot be equated with the speed of algorithm optimization. In order to compare the searching speed of each algorithm and make the comparison results reliable, fault section 16, which has little difference in the searching accuracy rate of the three algorithms in Table 1, is selected for data analysis. The specific setting of the program here is to run the three algorithms for 200 times, respectively. When the optimal value is iterated in an experiment, the experiment will be ended, and the time will be accumulated for the next experiment. The final comparison result is shown in Fig. 5.

According to the running time of the three algorithms in Fig. 5, the SPSO-GA algorithm constructed in this paper is about 1/3 longer than that of the PSO-GA algorithm. In contrast, the iteration time of the AF algorithm is more than twice as long as that of the PSO-GA algorithm. Therefore, in combination with Figs. 4 and 5, it can be concluded that under the condition of high accuracy of the three algorithms. However, AF requires fewer iterations, and its running time is the longest among the three algorithms, which is determined by the large computation amount of the AF algorithm itself. In addition, although the iteration speed of PSO-GA is fast, combined with the previous analysis, it can be seen that the optimization accuracy of this algorithm is the lowest. Therefore, overall, the algorithm constructed in this paper is still dominant.

## Conclusion

In this paper, we break away from the binary nature of fault coding in distribution networks and introduce a successive particle swarm coding approach. This method is then combined with the principles of genetic algorithms to form a new hybrid algorithm called SPSO-GA. The accuracy, fault tolerance, and optimization speed of SPSO-GA, AF, and PSO-GA in locating faults within the IEEE33 node distribution network were compared. The results indicate that the SPSO-GA hybrid algorithm proposed in this paper exhibits high optimization accuracy, stability, and fault tolerance across various fault conditions. Moreover, it demonstrates rapid execution speed and performs well even with a small number of particles. These advantages collectively position SPSO-GA as a superior choice compared to other algorithms.

Overall, the findings of this study offer new insights into addressing fault localization issues in multi-power-source structures, contributing to reducing operational costs and improving the stability of power networks. As technology advances and algorithms are optimized, these outcomes will serve as valuable references and foundational support for achieving more reliable, intelligent, and efficient power systems.

## Data Availability

The data that support the findings of this study are available on request from the corresponding author, Jiachun Li, upon reasonable request.
